# Interactions between the human milk oligosaccharide 2′-fucosyllactose and *Bifidobacterium longum* subspecies *infantis* in influencing systemic immune development and function in piglets

**DOI:** 10.3389/fnut.2024.1444594

**Published:** 2024-10-25

**Authors:** Victoria C. Daniels, Marcia H. Monaco, Johanna Hirvonen, Arthur C. Ouwehand, Henrik Max Jensen, Ratna Mukerjea, Niels Christensen, Markus J. Lehtinen, Ryan N. Dilger, Sharon M. Donovan

**Affiliations:** ^1^Division of Nutritional Sciences, University of Illinois, Urbana, IL, United States; ^2^Department of Food Science and Human Nutrition, University of Illinois, Urbana, IL, United States; ^3^IFF Health and Biosciences, Kantvik, Finland; ^4^IFF R&D—Enabling Technologies, Brabrand, Denmark; ^5^IFF Health and Biosciences, Saint Louis, MO, United States; ^6^Department of Animal Sciences, University of Illinois, Urbana, IL, United States

**Keywords:** *B. infantis* Bi-26, immune, cytokine, human milk oligosaccharides, 2-fucosyl-lactose

## Abstract

**Introduction:**

The oligosaccharide 2′-fucosyllactose (2′-FL) is a predominant component of human milk, serving as a prebiotic for gut microbiota and influencing immune development in infants. *Bifidobacterium longum* subspecies *infantis (B. infantis)* is a commensal bacterium found in breastfed infants. Both 2′-FL and a specific strain of *B. infantis*, Bi-26™, are commercially available. This study investigates the potential synbiotic relationship between 2′-FL and Bi-26™ on immune development.

**Methods:**

Two-day-old piglets (*n* = 53) were randomized in a 2 × 2 design, receiving either a commercial milk replacer *ad libitum* without (CON) or with 1.0 g/L 2′-FL (FL). Piglets in each diet were further randomized to receive either glycerol stock alone or Bi-26™ (10^9^ CFU) (BI and FLBI) orally once daily. On postnatal day (PND) 34/35, animals were euthanized, and blood was collected for serum cytokine analysis. Additionally, peripheral blood mononuclear cells (PBMCs) were isolated for *ex vivo* stimulation and flow cytometry analysis. Serum and *ex vivo* cytokines were analyzed using a multivariate model. All other outcomes were analyzed using a two-way ANOVA, considering prebiotic and probiotic fixed effects. The significance level was set at a *p* value <0.05, with trends reported for 0.05 < *p* < 0.1.

**Results:**

Immune cell populations in PBMCs were unaffected by the experimental treatment. However, serum interleukin (IL)-1RA, IL-1β, IL-12, and IL-18 were all higher (*p* < 0.05) in the FL group than in the CON group. In isolated PBMCs, lipopolysaccharide (LPS) stimulation resulted in higher IL-1RA and a trend for higher IFN-*γ* secretion in the FL group vs. the CON group.

**Conclusion:**

2′-FL stimulates a balanced cytokine profile in healthy piglets without changing immune cell populations. When immune cells are stimulated *ex vivo* with LPS, 2′-FL primes T-cells for a proinflammatory response, which is moderated by co-administration of Bi-26™.

## 1 Introduction

The newborn infants’ immune system is functionally naïve, leaving them vulnerable to pathogens. Morbidity and mortality are higher in non-breastfed infants (1), which is attributed to the bioactive components in human milk that stimulate immune maturation and development of the gut microbiome (2). Among these bioactive components are the multifunctional human milk oligosaccharides (HMOs), which serve as prebiotics and inhibitors of pathogen attachment to the epithelial lining (3). Infant formulas are virtually devoid of these glycans since they are in low concentration in bovine milk, which is the starting material for most infant formulas (3).

In human milk, 2′-fucosyllactose (2′-FL) is one of the most abundant HMOs in mothers who express the 2-fucosyltransferase gene (4). A small amount of 2′-FL is absorbed into the systemic circulation in human infants (5) and rats (6). There is evidence that 2′-FL modulates cytokine production and receptor signaling, but data are not entirely consistent. Co-culturing cord blood mononuclear cells with neutral HMOs had little effect on intracellular production of IFN-*γ*, IL-4, and IL-13, but acidic HMOs increased the production of these cytokines (7). Preclinical and clinical studies have demonstrated that 2′-FL alone directly impacts immune development and inflammation. Infants fed formula supplemented with 0.2 or 2.0 g/L 2′-FL had serum IL-1RA, IL-1β, IL-1*α*, tumor necrosis factor-α, and IL-6 concentrations that were not statistically different from those of breastfed infants (8). In human infant and mouse intestinal explants, 2′-FL decreased the severity of necrotizing enterocolitis by binding to the TLR4-myeloid differentiation factor 2-[MD2] complex, which prevented lipopolysaccharide (LPS) binding (9). Furthermore, 2′-FL ameliorated inflammatory response to rotavirus (RV) infection in the small intestine of rat pups and reduced IL-*β*, IL-6, and IFN-*γ* production at the peak of infection (10). In addition, in healthy suckling rat pups, an oral gavage of 2′-FL increased plasma IgG and IgA levels as well as T-helper cell populations in the mesenteric lymph nodes (MLNs) (11). Thus, 2′-FL has shown a range of immunomodulatory characteristics that may benefit infants.

*Bifidobacterium longum* subspecies *infantis (B. infantis)* is a common resident of the gut microbiota of breastfed, but not formula-fed infants. A body of literature also supports the ability of *B. infantis* to influence cytokine production and health outcomes. In human intestinal NEC models, *B. infantis* reduced markers of inflammation, such as IL-6 and IL-8 production, and decreased the upregulation of NFκB-related genes *in vitro* (12). In a clinical trial evaluating the enteric immunomodulatory effects of *B. infantis* EVC001, IL-8, IL-22, IL-1β, and IFN-*γ* were lower in infants receiving probiotic supplementation than in control infants only being breastfed (13). In addition, in a placebo-controlled, double-blind, randomized study in human infants, supplementation with *B. infantis* increased the IL-10/IL-12 ratio toward an anti-inflammatory effect (14). In a piglet model of *Staphylococcus aureus* infection, post-infection, *B. infantis* supplementation increased serum IL-10 level concentrations and decreased memory T-cell populations (15). Thus, *B. infantis* holds promise as an immunomodulatory probiotic supplement for human infants.

Recent evidence also supports a synbiotic potential for combined administration of 2′-FL and *B infantis*. In addition to direct effects on the immune system, 2′-FL stimulates the growth of *Bifidobacterium*, and *B. infantis* can use 2′-FL for growth and metabolite production (16, 17). Bi-26™ is a commercially available strain of *B. infantis* in the Danisco Global Culture Collection. Its genome reveals that Bi-26™ contains the enzymes necessary to digest 2′-FL. Utilization of 2′-FL by Bi-26™ produces a more diverse group of metabolites than those generated from lactose utilization (18). However, the potential symbiotic effects of Bi-26 and 2′-FL on immune function and development *in vivo* are not well studied.

Therefore, we aimed to better understand the individual and synbiotic effects of dietary 2′-FL and Bi-26™ on piglet systemic immune development. Because of their individual effects, we hypothesized that both 2′-FL and Bi-26™ would promote healthy development of systemic immunity. We further hypothesized that piglets exposed to the combination of 2′-FL and Bi-26™ would have lower levels of serum pro-inflammatory cytokines than CON or 2′-FL, but immune cells isolated from these piglets would demonstrate a more robust response to *ex vivo* LPS stimulation than cells from piglets fed either 2′-FL or Bi-26™ alone.

## 2 Materials and methods

### 2.1 Experimental design

The Institutional Animal Care and Use Committee at the University of Illinois approved all animal care and experimental procedures. The experimental design, dietary treatments, and probiotic administration were previously described by Daniels et al. (19). Briefly, naturally farrowed piglets (*n* = 63) from a commercial swine herd received colostrum from the sow for up to 2-day (PND2) after birth before being transported to a specialized neonatal pig rearing system at the Piglet Nutrition and Cognition Laboratory (PNCL) on the University of Illinois campus. Piglets were randomized into the four treatment groups in a 2 × 2 design. Two experimental milk replacer diets (CON and 2′-FL) were manufactured in powder form by TestDiet (Purina Mills, St. Louis, MO). The composition was identical to ProNurse (Land O’Lakes, North Arden Hills, MN) except that the CON diet had added lactose equal to the 2′-FL content in the test diet (1 g/L in the reconstituted formula). The 2′-FL (CARE4U®) was provided by International Flavors & Fragrances (New York, NY, United States). CARE4U® is a high purity (≥94%) infant-grade 2′-FL containing ≤300 EU endotoxins/g product. Liquid milk replacer was reconstituted daily at 20% solids and automatically delivered to piglets beginning at 1,000 h each day and ending at 0600 h the following day (i.e., *ad libitum* access to treatment diets over a 20-h feeding period each day). Milk disappearance was calculated per individual pig based on the initial and final reservoir weights collected before and after the daily feeding cycle. Piglets in the CON and 2′-FL diet groups were further randomized to probiotic or no probiotic, resulting in four experimental treatment groups (CON, FL, BI, and FLBI). Aliquots of the probiotic treatment (Bi-26™; Danisco Global Culture collection DGCC11473 Niebüll, Germany) at the dose of 10^9^ CFU/day were solubilized before the start of the study in bacteria glycerol stock (12.1% glycerol) and frozen until administration. The treatment was thawed and orally dosed daily. Piglets not receiving probiotic treatment received an equal dose of glycerol stock.

### 2.2 Sample collection

On PND 34 or 35, piglets were sedated with an intramuscular injection of Telazol® (Tiletamine HCl and Zolazepam HCl, 3.5 mg/kg BW each, Pfizer Animal Health, Fort Dodge, IA). Blood samples were collected by intracardiac puncture into heparin-laced vials (BD Biosciences, Franklin Lakes, NJ, United States) to isolate peripheral blood mononuclear cells (PBMCs). In addition, blood samples were collected and prepared as serum and or plasma by centrifugation. Piglets were then euthanized by an intracardiac injection of sodium pentobarbital (72 mg/kg BW; Fatal Plus; Vortech Pharmaceuticals, Dearborn, MI).

### 2.3 Immune cell isolation

Peripheral blood mononuclear cells were isolated on PND 34 or 35 following necropsy as previously described (20). Blood was initially diluted with DPBS (Dulbecco’s PBS, no Ca^++^, no Mg^++^, Life Technologies, Thermo Fisher, Carlsbad, CA) at a 1:1 ratio, then layered onto Ficoll-Paque Plus (GE Healthcare, Piscataway, NJ), and spun at 400 × *g* for 40 min at 20°C. PBMCs were collected from the gradient interface and washed three times in wash buffer (Hanks Buffered Salt Solution, no Ca^++^, no Mg^++^, Life Technologies) supplemented with 2% bovine serum albumin (BSA; Sigma-Aldrich, St. Louis, MO), 0.01 M EDTA (Sigma-Aldrich), 50 μg/mL Gentamycin (Life Technologies), 1,000 U/mL penicillin (Sigma-Aldrich), and 100 μg/mL streptomycin (10 mg/mL stock, Sigma-Aldrich). The remaining red blood cells in the pellet were lysed with lysis buffer (0.15 M of NH_4_Cl, 10 mM KHCO_3_, and 0.1 mM Na_2_EDTA). PBMCs were suspended in RPMI-1640 supplemented with 10% fetal bovine serum (FBS), 2 mM glutamine, 50 μg/mL gentamycin, 1 mM sodium pyruvate (Life Technologies), 20 mM HEPES (Life Technologies), and 20 mM 1,000 U/mL penicillin/100 μg/mL streptomycin prior to quantification. The number of viable cells was assessed with Countess® Automated Cell Counter (Life Technologies). Cells were then used for phenotypic cell identification by flow cytometry and *ex vivo* stimulation as described below.

### 2.4 Phenotypic identification of immune cells in PBMC

Phenotypes of PBMC subpopulations were analyzed by flow cytometry (BD™ LSRII, Biosciences) in the Roy J Carver Biotechnology Center Flow Cytometry facility at the University of Illinois. A panel of fluorescein (FITC) or Phycoerythrin (PE)-labeled mAbs was following a previously established staining procedure (20, 21). Briefly, T-lymphocytes were identified by mouse anti-pig CD4 (FITC, Clone 74-12-4) and mouse anti-pig CD8 (PE, Clone 76-2-11) antibodies (Southern Biotech, Birmingham, AL). B-lymphocytes were identified by mouse anti-pig SLA class II (FITC, Clone 2E9/13) and mouse anti-pig CD21 (PE, Clone BB6-11C6.9). A total of 10 μL of each antibody was added to 1 × 10^6^ cells from each sample. Staining procedures were carried out on ice, and the samples were protected from light when possible. In brief, all cells were initially incubated with 5 μg/mL of unlabeled mouse anti-pig CD16 (20 μL; AbD Serotec, Raleigh, NC) to prevent non-specific binding. Each well was blocked with 5% mouse serum (Southern Biotech) for 5 min each. After centrifugation, CD3 was added to the wells, incubated for 20 min (50 μL: CD3:PE-Cy5; Southern Biotech), and centrifuged again. CD4:FITC and CD8:PE were added or SLAII:FITC and CD21:PE (10 μL each) and incubated for an additional 15 min until centrifuged. Cells were washed with PBS/1% BSA/0.1% sodium azide and then fixed with 2% paraformaldehyde. Cells were assessed using an LSRII flow cytometer (BD™, Biosciences). The percentage of T-cell subpopulations and B-cell populations was determined using FCS 4 Express software (DeNovo Software; Glendale, CA). CD3^+^ events were considered T-cells. CD3^+^CD4^+^CD8^−^ events were considered T-helper cells, and CD3 + CD4-CD8+ and CD3 + CD4 + CD8+ were considered cytotoxic T and memory T-cells, respectively. B-cells were identified at CD21^+^ and SLA class II^+^ dual positive cells using SLAII:FITC- and CD21PE-labeled antibodies.

### 2.5 Serum cytokine concentrations

The concentrations of 13 cytokines, namely, granulocyte-macrophage colony-stimulating factor (GM-CSF), interferon-gamma (IFNγ), tumor necrosis factor-alpha (TNFα), interleukin (IL)-1α, IL-1RA, IL-1β, IL-2, IL-4, IL-6, IL-8, IL-10, IL-12, and IL-18 were measured using a Millipore (Milliplex) porcine cytokine array at the University of Illinois Flow Cytometry Center. When the value for a sample fell below the limit of detection (Supplementary Table 1), values were set to 0.

### 2.6 *Ex vivo* cell stimulation and cytokine production

Isolated PBMC were cultured to assess *ex vivo* cytokine secretion in response to LPS and phytohemagglutinin (PHA) stimulation as previously described (20, 21) Briefly, cells (2 × 10^5^ cells/well) were plated in 96-well plates in a final volume of 200 μL in complete RPMI and were maintained at 37°C under 5% CO_2_. Triplicate wells per cell type and experimental treatment were immediately stimulated with 2 μg/mL LPS and 2.5 μg/mL PHA, a lymphocyte activation and proliferation modulator. Cell culture supernatants were collected 72 h after culture initiation and frozen at −80°C until analyzed. Supernatants from the *ex vivo* assay were analyzed for 13 cytokines using a Millipore (Milliplex) pig cytokine array described for serum. In addition, IL-17 was analyzed using an ELISA kit (Raybiotech, Norcross, GA) according to the manufacturer’s instructions, with the exception that PBS was used as the basis for the assay buffers.

### 2.7 Statistical analyses

All data, except for the cytokine data, were analyzed using SAS version 9.4 (SAS Institute Inc., Cary, NC) using the PROC MIXED procedure. Immunoglobulin and immune cell data were analyzed using a two-way ANOVA. The statistical model was nested for random effects with replicate nested within sow. Both models included fixed effects of diet (prebiotic), probiotics, and the interaction between diet and probiotics with Dunnett’s *post hoc* test to compare the statistical differences between the control and experimental treatment groups. If samples were ± 3 standard deviations from the mean, they were excluded as outliers. Significance was defined at a *p* value <0.05 and trends are reported as a *p* value <0.10. Data are presented as means ± standard error of the mean (SEM) unless otherwise stated.

Serum and *ex vivo* cytokine concentrations were analyzed using a PROC LCA model using SAS version 9.4 adapted to the respective design. The design for serum samples was a basic design with a split-plot structure. Whole plots were characterized by random effects of sow ID nested within the cohort. Split plots were characterized by piglet ID with the fixed effect of diet. The diet was composed as a 2-by-2 factorial from factors Bi-26™ and 2′-FL. The response is multivariate with manifestations of each cytokine (IFN-*γ*, IL-1α, IL-1β, IL-1RA, IL-2, IL-4, IL-6, IL-8, IL-10, IL-12, and IL-18). The design for the *ex vivo* analyses was a one-split of the basic design, by a treatment (unstimulated, LPS, PHA) for PBMC. Whole plots were characterized by random effects of sow ID nested within the cohort. Split plots were characterized by piglet ID with a fixed effect of diet. The diet was composed as a 2-by-2 factorial from factors Bi-26™ and 2′-FL. Split–split plots were characterized by random residual with nested fixed effect of treatment interaction between treatment and diet.

## 3 Results

### 3.1 Serum immunoglobulin and cytokine concentrations

No differences were found in serum IgG or IgM. Serum IL-1RA, IL-1β, IL-2, and IL-18 were all significantly higher in FL but not BI alone or FLBI than in CON (Table 1). Serum IL-12 was greater in FL than CON and FLBI but not BI alone. There were trends (*p* < 0.1) for IL-1α, IL-4, IL-6, and IL-10 to also be affected by diet or the interaction of diet and probiotics. Serum IFN-*γ* and IL-8 were unaffected by experimental treatment (Table 2). Serum IL-17A was below the level of detection.

**Table 1:** Serum immunoglobulin concentrations (mg/mL).

Treatment	IgG	IgM
CON	4.88 ± 0.66	0.84 ± 0.11
FL	4.61 ± 0.77	0.73 ± 0.09
BI	4.64 ± 0.52	0.72 ± 0.07
FLBI	6.26 ± 0.45	0.86 ± 0.10
*p* values		
Diet	0.7529	0.0.5956
Probiotic	0.7280	0.0.4991
Interaction	0.8828	0.0.7462

Data are expressed as means ± SEM. *n* = 9–12.

BI, control diet + 10^9^ CFU Bi-26™/day; CON, Control diet; FL, Control formula + 1.0 g/L 2′-FL; FLBI, Control formula + 1.0 g/L 2′-FL + 10^9^ CFU Bi-26™/day; Ig, Immunoglobulin; SEM, Standard error of the mean.

**Table 2:** Serum cytokine concentrations (pg/mL).

					*p* value		
	CON	FL	BI	FLBI	Diet	Probiotic	Interaction
IFN-*γ*	8,032 ± 2,729	7,265 ± 2,493	6,396 ± 2,506	8,863 ± 2,860	0.825	0.896	0.219
IL-1α	28.1 ± 17.2	73.6 ± 22.7	51.3 ± 21.6	46.6 ± 21.7	0.077	0.976	0.112
IL-1β	52.2 ± 48.5^a^	236.1 ± 84.3^b^	168.0 ± 79.1^ab^	129.7 ± 73.7^ab^	0.076	0.952	0.043
IL-1RA	94.5 ± 47.3^a^	274.1 ± 64.4^b^	192.4 ± 62.3^ab^	133.6 ± 56.4^ab^	0.053	0.803	0.034
IL-2	32.7 ± 40.7^a^	301.3 ± 98.2^b^	152.2 ± 82.2^ab^	129.3 ± 79.2^ab^	0.091	0.944	0.039
IL-4	96.7 ± 121.2	795.2 ± 282.1	530.5 ± 261.0	354.8 ± 229.0	0.099	0.968	0.091
IL-6	53.2 ± 34.4	175.8 ± 50.7	94.0 ± 43.3	88.4 ± 43.4	0.103	0.960	0.067
IL-8	228.2 ± 48.4	252.8 ± 46.7	263.9 ± 50.5	271.9 ± 52.7	0.575	0.976	0.984
IL-10	107.4 ± 89.9	490.9 ± 155.2	353.6 ± 149.6	269.5 ± 139.8	0.087	0.976	0.069
IL-12	439.7 ± 53.2^a^	552.0 ± 49.7^b^	550.1 ± 52.6^abc^	461.0 ± 53.8^ac^	0.139	0.652	0.005
IL-18	447.5 ± 196.6^a^	1,078 ± 244.3^b^	923.0 ± 252.3^ab^	731.5 ± 243.7^ab^	0.076	0.992	0.044

Data are expressed as means ± SEM. *n* = 9–12. Different letter superscripts indicate differences between groups at *p* ≤ 0.05.

BI, Control diet + 10^9^ CFU Bi-26™/day; CON, Control diet; FL, Control formula + 1.0 g/L 2′-FL; FLBI, Control formula + 1.0 g/L 2′-FL + 10^9^ CFU Bi-26™/day; IFN, Interferon; IL, Interleukin; PND, Postnatal day; SEM, Standard error of the mean; TNF, Tumor necrosis factor.

### 3.2 PBMC immune cell populations

No treatment effect was observed in T-cell subpopulations or B-cell populations in PBMC (Table 3).

**Table 3:** Immune cell populations in peripheral blood mononuclear cells.

					*p* value		
	CON	BI	FL	FLBI	Diet	Probiotic	Interaction
T-cells (% CD3^+^)	52.9 ± 3.69	54.8 ± 3.25	58.1 ± 0.92	55.1 ± 1.92	0.2044	0.6596	0.7493
Helper CD4^+^	11.4 ± 1.29	10.7 ± 1.11	9.3 ± 1.04	12.0 ± 1.04	0.9775	0.9194	0.4175
Cytotoxic CD8^+^	15.9 ± 2.39	14.6 ± 1.99	14.1 ± 1.41	13.8 ± 1.41	0.4935	0.8872	0.3323
Memory CD4^+^ CD8^+^	4.29 ± 0.82	4.01 ± 0.49	3.83 ± 0.83	3.90 ± 0.72	0.9296	0.9003	0.7979
B-cells (% MHCII^+^CD21^+^)	52.9 ± 3.69	54.8 ± 3.25	58.1 ± 0.92	55.1 ± 1.92	0.2044	0.6596	0.7493

Data are expressed as means ± SEM. *n* = 9–12.

BI, Control diet + 10^9^ CFU Bi-26™/day; CD, Cluster differentiation; CON, Control diet; FL, Control formula + 1.0 g/L 2′-FL; FLBI, Control formula + 1.0 g/L 2′-FL + 10^9^ CFU Bi-26™/day; PBMC, Peripheral blood mononuclear cells; SEM, Standard error of the mean.

### 3.3 PBMC *ex vivo* cytokine secretion

To investigate the impact of *in vivo* exposure to 2′-FL or Bi-26™ on cytokine secretion in response to LPS or PHA stimulation, the secretion of cytokines into the media was compared between unstimulated to stimulated PBMCs (Table 4). PBMC isolated from piglets fed FLBI stimulated with LPS *ex vivo* secreted higher concentrations of IL-1RA than CON (*p* ≤ 0.05) (Table 4). There was a trend (*p* < 0.1) for the effect of 2′-FL on IFN-*γ* secretion. There was also a trend (*p* < 0.1) for the effect of 2′-FL on IL-6 secretion. No other cytokines were affected by experimental treatment with stimulation from LPS or PHA compared to CON.

**Table 4:** Cytokine concentrations (pg/mL) in conditioned media from *ex vivo* stimulated peripheral blood mononuclear cells.

					*p* value		
	CON	FL	BI	FLBI	Diet	Probiotic	Interaction
IFN-γ							
Unstimulated	22.5 ± 0.77	20.2 ± 0.60	11.3 ± 0.27	5.92 ± 0.11	0.841	0.208	0.944
Stimulated	23.6 ± 0.82	166.6 ± 12.8	43.0 ± 1.95	48.5 ± 2.4	0.072	0.317	0.082
Fold-stimulation	1.05	8.32	3.80	8.20	0.121	0.834	0.165
IL-1RA							
Unstimulated	366.8 ± 7.26	453.0 ± 9.34	450.9 ± 9.71	813.7 ± 23.9	0.407	0.897	0.841
Stimulated	777.7 ± 22.1 ^a^	1,145 ± 36.7 ^a^	1,042 ± 33.4 ^a^	1,677 ± 68.5 ^b^	0.050	0.204	0.484
Fold-stimulation	2.12	2.53	2.31	2.06	0.136	0.070	0.509
IL-6							
Unstimulated	114.9 ± 3.66	166.0 ± 5.71	74.4 ± 1.88	25.2 ± 0.39	0.944	0.453	0.749
Stimulated	311.3 ± 15.7	643.3 ± 39.7	486.3 ± 29.1	504.7 ± 31.9	0.097	0.447	0.674
Fold-stimulation	2.71	3.87	6.54	20.1	0.089	0.112	0.430

Data are expressed as means ± SEM. *n* = 9–12. Different letter superscripts indicate differences between groups at *p* ≤ 0.05.

BI, Control diet + 10^9^ CFU Bi-26™/day; CON, Control diet; FL, Control formula + 1.0 g/L 2′-FL; FLBI, Control formula + 1.0 g/L 2′-FL + 10^9^ CFU Bi-26™/day; IFN, Interferon; IL, Interleukin; PND, Postnatal day; SEM, Standard error of the mean.

## 4 Discussion

Oligosaccharides in milk play an important role in modulating the development of the neonatal immune system directly and indirectly through altering the gut microbiota (22). A recent study by Rosa et al. demonstrated the direct effects of HMO in the absence of microbiota (23). Germ-free 1-day-old mice were gavaged daily with pooled HMOs (15 mg) for 7 or 14 days. Samples were collected on days 28, 35, or 50. HMO-administration induced differences in immune cell populations, antibody-secreting cells, and intestinal tissue gene expression in germ-free animals. Cytokine secretion was not measured in that study. Given that pooled HMOs were administered, it is not possible to attribute outcomes to specific individuals or categories of HMOs.

Herein, the individual and combined effects of 2′-FL and *B. infantis* were studied for the following reasons. Supplementing infant formula with all HMOs at the current time is not feasible. 2′-FL is a predominant HMO in the milk of secretor mothers (4), it has been supplemented to infant formulae for several years with no adverse effects (24) and has documented effects on infant immunity (8). *B. infantis* was studied as formula-fed infants are devoid of *B. infantis*, a predominant bifidobacterial strain in breastfed infants, it can use 2′-FL and has immunomodulatory effects and has documented effects on infant immunity (16, 18). Given the demonstrated benefits of supplemental 2′-FL (8, 25) and *B. infantis* (13, 26), documented in previous clinical trials, and their potential for symbiotic activity, we investigated the individual and combined effects of dietary 2′-FL at 1 g/L and Bi-26™ at 10^9^ CFU on immune development and function in piglets. Our findings show the effects of 2′-FL systemic immunity and potential interactive effects when supplemented with Bi-26™.

We found no effect of dietary 2′-FL and/or Bi-26™ on circulating immune cell populations; all levels were within the range previously reported in our laboratory for healthy piglets (27, 28), suggesting that there was no impact on T-cell or B-cell percentages. In a clinical trial of 2′-FL supplementation, circulating CD4^+^ and CD8^+^ T-lymphocyte populations were similar between breastfed infants and infants fed formula supplemented with 2′-FL (8). Since we saw no differences in T-cell subsets or B-cells in PBMC, 2′-FL could be affecting innate immunity. Although dendritic cells make up a small percentage of circulating immune cells, they present antigens to T-cells, leading to the upregulation of cytokines to recruit immune cells to clear the pathogen (29). IL-10 and IL-6 are upregulated in dendritic cells treated with HMOs isolated from human milk (30). Increased dendritic cell activity could recruit T-helper cells, which could explain why the serum cytokines were increased in our study; however, more research is required to establish the precise mechanism at hand.

A key finding of the present study was that 2′-FL increased the concentrations of IL-1RA, IL-1β, IL-2, IL-12, and IL-18 in serum compared to control piglets. The group receiving both 2′-FL and Bi-26™ did not differ from the control group, suggesting that the co-administration of Bi-26™ may moderate the effect of 2′-FL impact on serum cytokines. Studies in human infants have shown that breastfeeding is associated with lower circulating concentrations of proinflammatory cytokines (8, 31). In a clinical trial of 2′-FL supplementation, infants fed formula alone had higher IL-1RA, IL-1β, IL-1*α*, tumor necrosis factor-α, and IL-6 than infants fed formula supplemented with 0.2 g/L and 2.0 g/L 2′-FL. Infants supplemented with 2′-FL had inflammatory cytokine concentrations similar to that of breastfed infants (8). Similar observations have been made in rat studies, with 2′-FL lowering IL-1β, IL-4, IL-6, IL-12, IFN-*γ*, and TNF-α in the small intestine (11) and serum IL-18 in male rats compared to control (32). The study of Goehring et al. (8) demonstrated similar effects of 0.2 g/L and 2.0 g/L on serum cytokines, supporting a lack of dose dependency on cytokine concentrations (8); so, it is unlikely we would have seen lower cytokine concentrations with a higher or lower dose. Intact HMOs can be absorbed into the blood, and it is likely that immune cells are directly influenced in circulation.

In addition to measuring circulating cytokines, we assessed whether *in vivo* exposure to 2′-FL and/or Bi-26™ would impact cytokine secretion by immune cells isolated from blood (PBMC) when exposed to LPS or PHA *ex vivo*. Cytokines are predominantly produced by T-helper cells and macrophages (29). Although, in the present study, T-cell and B-cell populations in PBMC were unaffected by 2′-FL and Bi-26™, these supplements impacted immune response to LPS *ex vivo.* We found that PBMC, isolated from piglets fed 2′-FL alone, demonstrated increased secretion of IL-1RA, after LPS stimulation. In addition, *in vivo* exposure to 2′-FL tended to increase IFN-*γ* and IL-6 secretion. IFN-γ induces a wide array of immune responses. It can promote macrophage activation, facilitate antiviral and antibacterial activities, increase antigen presentation, and influence activation of the innate immune system (33) while IL-1RA is an inhibitor of pro-inflammatory IL-1 (34). LPS is a TLR-4 agonist, which leads to the production of IL-1, so the increase in IL-1RA and IL-6 could be a response to ameliorate inflammation caused by LPS challenge since 2′-FL and *B infants* have been shown to have anti-inflammatory effects. These results may be due to receptors that recognize HMOs, such as galectins and TLRs. Since HMOs have been found in the blood (6, 35), binding of HMOs to galectins in the blood could result in the regulation of TLR expression. In addition, 2′-FL has been shown to bind directly to TLR-4 *in silico* and modulate response to LPS by reducing TLR-4 expression in mice (9). An *in vitro* model of necrotizing enterocolitis in immature human enterocytes, *B. infantis* (ATCC 15697) conditioned medium reduced expression of inflammatory markers, TLR-2 and TLR-4, as well as concentrations of IL-6 and IL-8 (36). In a piglet model of *Staphylococcus aureus* infection, piglets supplemented with *B. infantis* (ATCC 15697) at 3 × 10^9^ CFU/day had increased serum IL-10, potentially as an attempt by the immune system to reduce the inflammatory response (15). Although no increase in IL-10 was observed following LPS stimulation, an increase in IL-1RA secretion by PBMC from piglets in the combined group was observed, suggesting that 2′-FL and Bi-26™ may still exhibit an anti-inflammatory effect. In addition, PBMC stimulated with PHA showed no effect of experimental treatment compared to the control formula. PHA is a lectin that requires monocytes, which are antigen-presenting cells, to induce T-cell proliferation (37). Our data suggests that neither 2′-FL nor Bi-26™ enhances T-cell proliferation in response to PHA compared to the control formula. Our study showed that dietary exposure to 2′-FL elicited a more robust cytokine response to the LPS challenge than from cells isolated from piglets fed the control formula. This could translate to improved immune response in infants receiving these HMOs.

Piglets were not immunologically challenged *in vivo*, therefore this study may not be translational to infants enduring infection. Although *ex vivo* stimulation shows a robust response to LPS stimulation, *ex vivo* conditions do not exactly mirror *in vivo* inflammation (38); therefore, an *in vivo* immune challenge could better evaluate the potential protective effects of 2′-FL and Bi-26™. A limitation of the study is that all immune analyses were from samples taken at a single time point at the end of the study, which limits our ability to evaluate the influence 2′-FL and Bi-26™ have on early immune development.

To summarize, human milk is the ideal feeding mode for infants; however, the World Health Organization (WHO) reports that globally, 66% of infants are not exclusively breastfeeding by 6 months of age, in accordance with the recommendations from 2015–2020 (39). As a result, many families must rely on infant formula during this critical period of development. While 2′-FL and *B. infantis* are commercially available, this is the first study to our knowledge to assess their combined effect on immune development in the piglet model. We have shown that the addition of 2′-FL and/or Bi-26™ could provide formula-fed infants with certain protective benefits associated with bioactive components that are typically lacking in infant formulae. Future investigations are needed to evaluate the effects of 2′-FL and Bi-26™ in response to immune stimuli during development and to explore the underlying mechanisms of these immune responses. This study builds upon previous observations demonstrating that supplementation of formula with 2′-FL and/or Bi-26™ is well tolerated and supports normal growth of piglets (19). Additionally, supplementation with 2′-FL and/or Bi-26 has been associated with changes in exploratory behaviors in novel object recognition tests, as well as changes to brain macro/micro-structures (40). Taken together, these findings support the multifunctional activities of 2′-FL and/or Bi-26™ in neonates.

## Data Availability

The raw data supporting the conclusions of this article will be made available by the authors, without undue reservation.
