# Supplementary material for: Interactions between the human milk oligosaccharide 2′-fucosyllactose and Bifidobacterium longum subspecies infantis in influencing systemic immune development and function in piglets
Source: Front Nutr. 2024 Oct 25;11:1444594. doi: 10.3389/fnut.2024.1444594 (PMC11543533; doi:10.3389/fnut.2024.1444594)
Supplement: Supplementary file 1 [file Table_1.DOCX]

Supplementary Material

The Human Milk Oligosaccharide 2′-Fucosyllactose and *Bifidobacterium longum subspecies infantis* Interact to Influence Systemic Immune Development and Function in the Piglet

Victoria C. Daniels, Marcia H. Monaco, Johanna Hirvonen, Arthur C. Ouwehand, Henrik Max Jensen, Ratna Mukerjea, Ryan N. Dilger, Sharon M. Donovan

*** Correspondence:** Corresponding Author: [sdonovan@illinois.edu](mailto:sdonovan@illinois.edu)

**Supplemental Table 1.** Limit of Detection for Commercial Multiplex Assay† used to Measure Cytokines in Serum and *Ex Vivo* Cell Supernatants.

| **Cytokine** | **Limit of Detection (ng/mL)** |
| --- | --- |
| GM-SCF | 0.02 |
| IFN-γ | 0.042 |
| IL-1α | 0.005 |
| IL-1β | 0.042 |
| IL-1RA | 0.01 |
| IL-2 | 0.008 |
| IL-4 | 0.02 |
| IL-6 | 0.009 |
| IL-8 | 0.005 |
| IL-10 | 0.009 |
| IL-12 | 0.005 |
| IL-18 | 0.012 |
| TNF-α | 0.006 |
| †Millipore Sigma. Abbreviations: GM-CSF, granulocyte-macrophage colony-stimulating-factor; IFN, interferon; IL, interleukin; TNF, tumor necrosis factor | |
